# Reactive and anticipatory looking in 6-month-old infants during a visual expectation paradigm

**DOI:** 10.1016/j.dib.2017.08.049

**Published:** 2017-09-04

**Authors:** Jeffry Quan, Jean-François Bureau, Adam B. Abdul Malik, Johnny Wong, Anne Rifkin-Graboi

**Affiliations:** a University of Ottawa, Canada; b Singapore Institute for Clinical Sciences, Singapore

## Abstract

This article presents data from 278 six-month-old infants who completed a visual expectation paradigm in which audiovisual stimuli were first presented randomly (random phase), and then in a spatial pattern (pattern phase). Infants’ eye gaze behaviour was tracked with a 60 Hz Tobii eye-tracker in order to measure two types of looking behaviour: reactive looking (i.e., latency to shift eye gaze in reaction to the appearance of stimuli) and anticipatory looking (i.e., percentage of time spent looking at the location where the next stimulus is about to appear during the inter-stimulus interval). Data pertaining to missing data and task order effects are presented. Further analyses show that infants’ reactive looking was faster in the pattern phase, compared to the random phase, and their anticipatory looking increased from random to pattern phases. Within the pattern phase, infants’ reactive looking showed a quadratic trend, with reactive looking time latencies peaking in the middle portion of the phase. Similarly, within the pattern phase, infants’ anticipatory looking also showed a quadratic trend, with anticipatory looking peaking during the middle portion of the phase.

**Specifications Table**

**Table:** 

Subject area	*Psychology*
More specific subject area	*Cognitive developmental psychology*
Type of data	*Descriptive statistics, figures*
How data was acquired	*Tobii eye-tracker, 60 Hz*
Data format	*Analyzed*
Experimental factors	*n/a*
Experimental features	*Behavioral analysis of 6-month-old infants who completed a visual expectation paradigm*
Data source location	*Singapore Institute for Clinical Sciences, Singapore*
Data accessibility	*Data is presented in the article*

**Value of the data**

• The data demonstrate how 6-month-old infants’ looking behaviours are influenced by the presentation of spatially patterned stimuli (compared to randomly presented stimuli)
• The data show temporal shifts in reactive and anticipatory looking behaviour as audiovisual stimuli are presented in a spatial pattern
• The data can serve as a reference point against which reactive and anticipatory looking from other time points in development can be compared

## 1 Data

Among the 439 infants to whom a visual expectation task was administered, 150 cases were excluded due to missing eye-tracker data resulting from either E-Prime video loading difficulties or E-Prime-Tobii interface difficulties, and 11 cases were lost due to human error. Thus, in total, 278 provided data for any portion of the task, and the data presented below describes measures of looking behaviour for this sample of infants.

### 1.1 Task order

The visual expectation paradigm was administered in counter-balanced order with two other eye-tracking tasks; however, the order in which the visual expectation task was administered was not significantly associated with any of the three looking behaviours measured. That is, task order was not related to reactive looking to random stimuli [F(2,256)=2.06, p=.130], reactive looking to patterned stimuli [F(2,251)=0.84, p=.432], nor anticipatory looking [F(2,269)=2.30, p=.102]. Likewise, task order was not significantly associated with the number of missing trials (either due to the infant looking away or due to the eye-tracker's inability to detect the infant's gaze) for reactive looking to random stimuli [*F*(2,275)=0.07, *p=*.931], nor reactive looking to patterned stimuli [*F*(2,275)=2.12, *p*=.122]. It was, however, marginally associated with the number of missing trials for anticipatory looking [*F*(2,275)=2.87, *p*=.059], with those who were administered the task first having marginally fewer missing trials (*M*=10.6, SD=8.2) than those administered the task second [M= 13.4, SD=10.3; *t*(180)=−1.96, *p*=.051] and significantly fewer missing trials than those administered the task last [*M*=13.6, SD=9.8; *t*(185)=−2.18, *p*=.030].

### 1.2 Performance across random and pattern phases

**Fig. 1:**
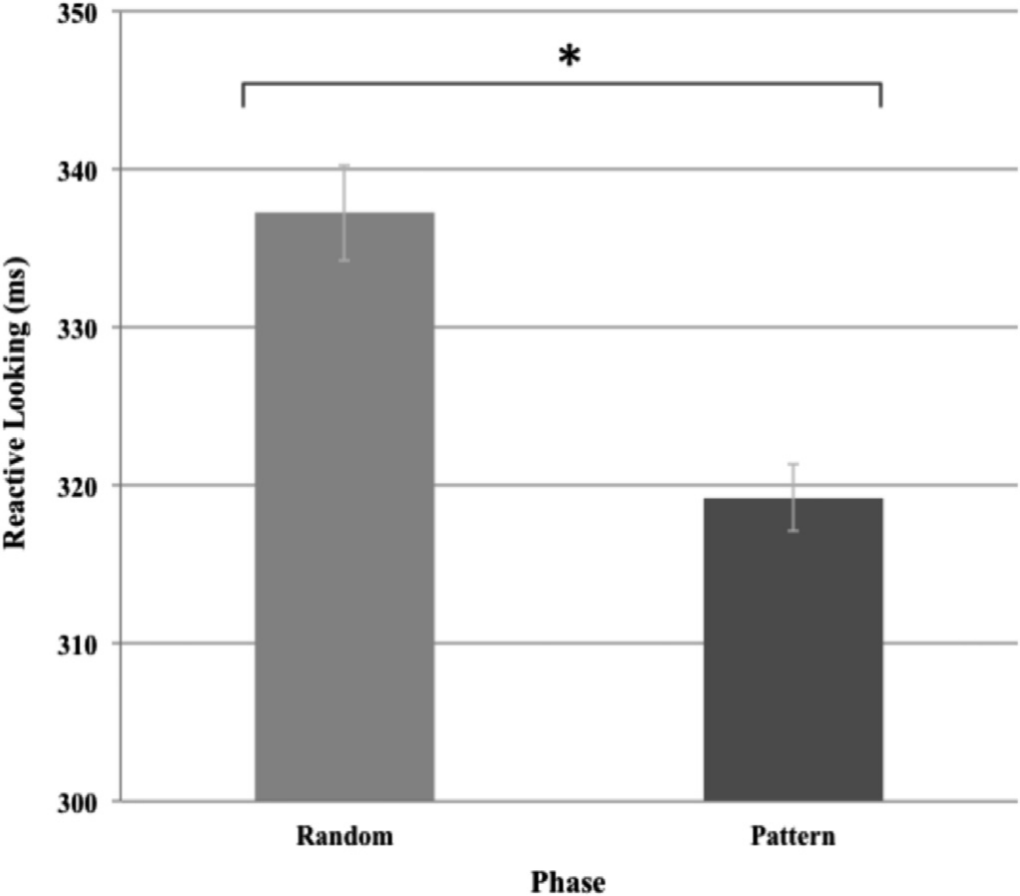
Reactive looking across random and pattern phases.

**Fig. 2:**
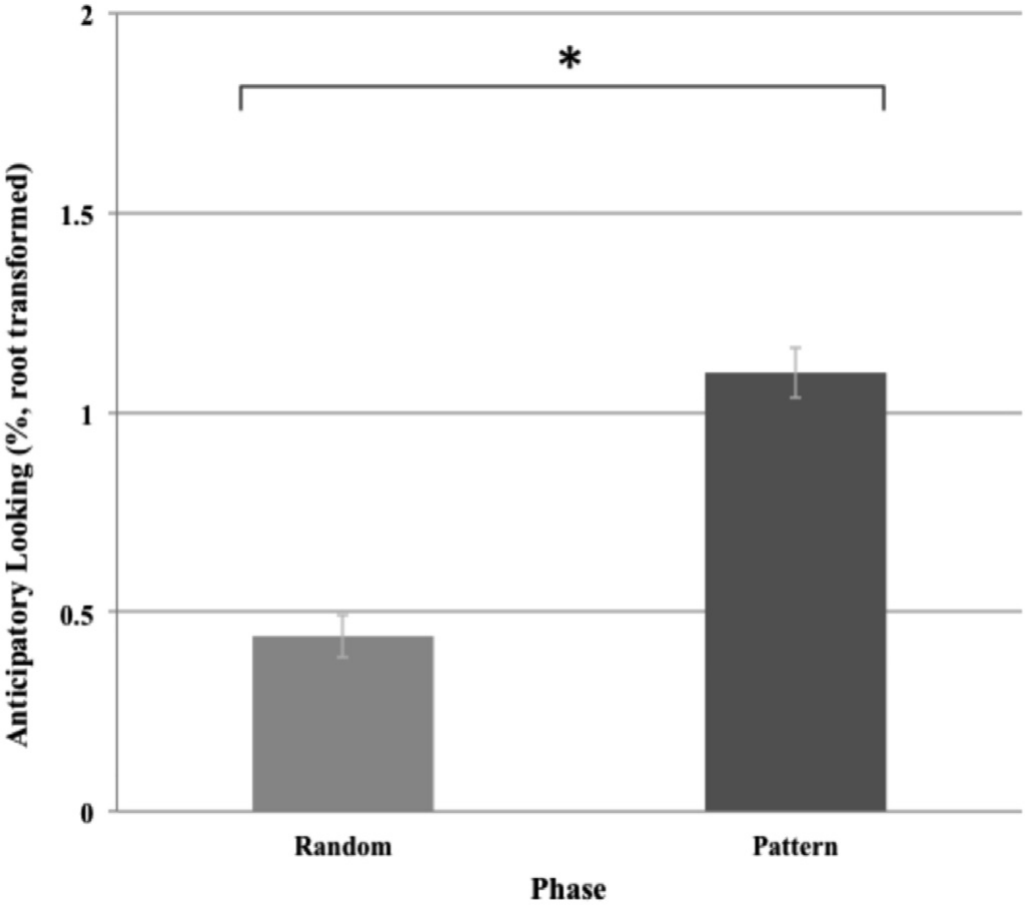
Anticipatory looking across random and pattern phases.

As shown in Fig. 1, reactive looking to patterned stimuli was significantly faster than reactive looking to random stimuli [t(244)=−5.39, p < .001]. As shown in Fig. 2, anticipatory looking was significantly greater than pre-stimulus looking during random stimuli [t(265)=9.28, p < .001].

### 1.3 Performance within pattern phase

**Fig. 3:**
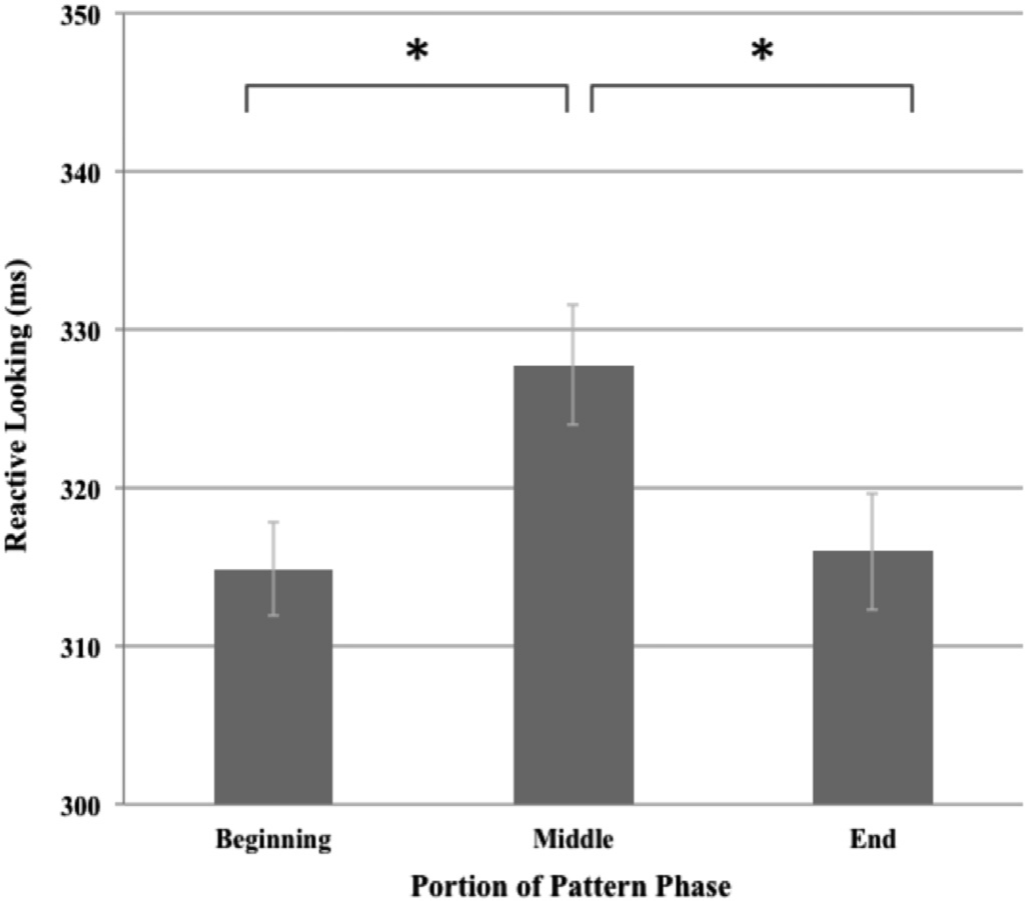
Reactive looking across beginning, middle, and end of pattern phase.

As shown in Fig. 3, reactive looking during the beginning, middle, and end (i.e., trials 1–20, 21–40, and 41–60) of the pattern phase exhibited a significant quadratic trend [F(1,209)=10.54, p=.001]. Pairwise comparisons showed that reactive looking was significantly slower during the middle of the pattern phase as compared to the beginning [t(209)=3.02, p=.003] and end [t(209)=2.56, p=.011], while reactive looking during the beginning and end of the pattern phase did not differ significantly [t(209)=−0.23, p=.816].

**Fig. 4:**
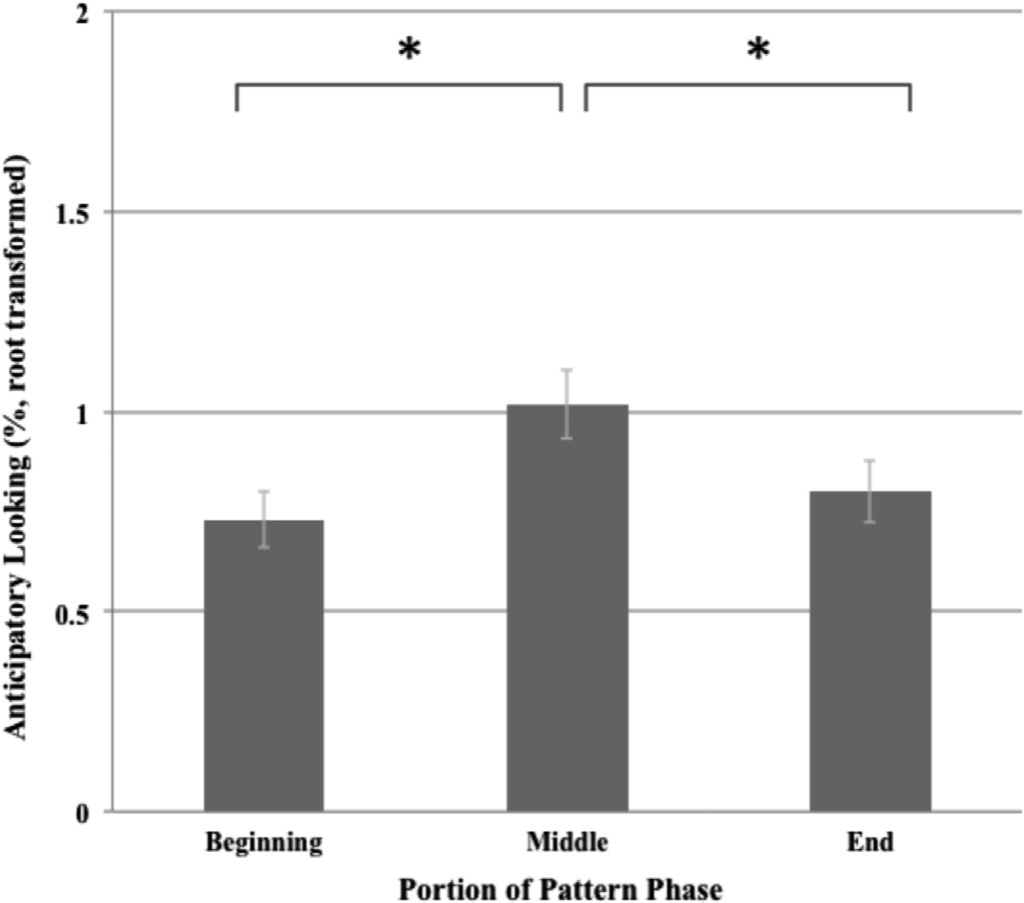
Anticipatory looking across beginning, middle, and end of pattern phase.

As shown in Fig. 4, anticipatory looking (root transformed) during the beginning. middle, and end of the pattern phase also exhibited a significant quadratic trend [F(1,240)=8.35, p=.004]. Pairwise comparisons showed that anticipatory looking during the pattern phase was significantly greater during the middle as compared to both the beginning [t(240)=2.93, p=.004] and end [t(240)=2.23, p=.028], while anticipatory looking during the beginning and end of the pattern phase did not differ significantly [t(240)=−0.66, p=.506].

## 2 Experimental design, materials and methods

Data were acquired via a Tobii eyetracker, and the Visual Expectation task was presented in a counter-balanced order with two other eye tracking tasks (relational binding, as described in [2] and habituation followed by a visual paired comparison, as described in [6]), but occurred after the encoding portion of a deferred memory task and before an electrophysiology task, a parent-child behavioral observation task, and the retrieval portion of the deferred memory task (see Cai et al. [1] for a more complete description of this laboratory visit and [4] for work examining this Visual Expectation task in relation to parenting behavior, also examined during the laboratory session).

Infants were seated on their parent's lap or in a high chair in front of a Tobii monitor, which was set at a sampling rate of 60 Hz. Infants were seated 60 cm away from the eye-tracker and the screen angle was adjusted via an Ergotron LX extension arm to increase feasibility. Calibration was not initiated until cameras detected the infants’ corneal reflection, which is generally optimal when the gaze angle does not exceed 42°. Infants were then shown a series of looming balls that appeared at the four corners and the center of the screen in sequence, and calibration accuracy was checked (with no more than two of five locations allowed to exceed reasonable error) and repeated if necessary.

Similar to the visual expectation paradigms used by Tamis-LeMonda and McClure [7] and Voelker et al. [8], infants were shown a series of 700 ms video clips with a 700 ms inter-stimulus-interval. Clips were presented with E-Prime software (Psychology Software Tools, Pittsburgh, PA) and took up 35% of the 17-inch monitor, centered on either the left or right side of the screen. Video clips were taken from musical episodes of children's television programs and randomized to create an ordered list common to all participants. The first 18 of these clips were presented randomly on either the left or right side of the screen, known as the random phase, and this was followed by a pattern phase, consisting of 60 clips appearing in an assigned order (i.e., left-left-right or right-right-left) on the monitor. An attention grabber was inserted after the first 18 clips of the random phase, and then after every 15 trials of the pattern phase.

Three Areas of Interest (AOIs) were defined: the background of the screen and the entirety of the left and right locations where the stimuli appeared. When an infant looked at any of these locations for at least 150 ms, the look was considered a “fixation” [5] and was considered in the calculation of three looking variables of interest: (1) reactive looking to random stimuli; (2) reactive looking to patterned stimuli; (3) and anticipatory looking. In cases of missing looking behavior data that did not exceed 150 ms, the location of looking behavior data both before and after the gap were examined to determine their consistency. When looking was found to be at the same location before and after the gap, the missing data were interpolated with this information, given that it would have been impossible for the infant to shift their gaze away from the location and back onto it within this short period [3]. If the resultant look exceeded 150 ms, it was then included in the calculation of the relevant visual expectation variable.

From the eye-tracking data, three primary variables of interest were calculated:

### 2.1 Reactive looking to random stimuli

An index of relatively pure reactive looking, or exogenously driven orienting, was calculated as the mean-time, in milliseconds, taken to fixate on stimuli presented across random trials. To ensure that values were not influenced by perseveration, data were only included from trials in which the stimulus did not appear on the same side of the screen as in the preceding trial (*M*=10.1 trials, *SD*=2.0, across all infants administered the visual expectation paradigm). As it takes up to 133 ms for an infant to physically shift his/her gaze in reaction to a stimulus [3], we then excluded data from trials in which the first fixation on the correct AOI occurred within 133 ms of stimulus onset, as these shifts could not have been reactive in nature. Thus, after accounting for trials during which there was no reactive look to the stimulus and trials for which gaze data were missing (either due to the infant looking away or due to the eye-tracker's inability to detect the infant's gaze), this variable included, on average, 4.4 (*SD*=2.6) valid trials across all infants who were administered the visual expectation paradigm.

### 2.2 Reactive looking to patterned stimuli

An index of pattern-influenced reactive looking was calculated as the mean-time, in milliseconds, prior to fixating on stimuli presented across pattern trials. In addition to exogenous (i.e., reflexive, stimulus-driven) influences, this reactive looking variable may also be influenced by endogenous expectations concerning where the stimuli is likely to occur. To avoid potential influences of perseveration, and because our pattern of stimuli appeared in either a left-left-right or right-right-left spatial pattern, this variable excluded trials in which the stimulus appeared at the same location as the one preceding it. Thus, this was a measure of how quickly infants looked reactively to stimuli after they appeared on the opposite side of the screen during the pattern phase of the task. Following the same logic as discussed above, we excluded trials in which looking towards the stimulus occurred within 133 ms of stimulus onset.

### 2.3 Anticipatory looking

An index of anticipatory looking was calculated as the mean amount of time spent looking at the correct location of the next stimulus in the pattern during the inter-stimulus intervals (ISI) of pattern trials, as a percentage of total time spent looking at the screen during this period. Similar to our reactive looking variable, to avoid the potential influence of perseveration, this variable excluded ISIs for trials in which the next stimulus appeared at the same location as the stimulus preceding it. Because this variable was positively skewed, a square-root transformation was applied. As discussed in the Statistical Analyses section below, a “pre-stimulus looking during random stimuli” variable was similarly calculated over random trials, which was to be entered as a control variable, thereby ensuring that our anticipatory looking variable reflected a form of executive attention, with the effects of attentional orienting removed. Pre-stimulus looking during random stimuli was calculated in an identical manner to the anticipatory looking, including the omission of looking behavior for trials occurring on the same side of the screen as the preceding trial.
